# Tungsten–SiO_2_–Based Planar Field Emission Microtriodes with Different Electrode Topologies

**DOI:** 10.3390/ma16175781

**Published:** 2023-08-24

**Authors:** Liga Avotina, Liga Bikse, Yuri Dekhtyar, Annija Elizabete Goldmane, Gunta Kizane, Aleksei Muhin, Marina Romanova, Krisjanis Smits, Hermanis Sorokins, Aleksandr Vilken, Aleksandrs Zaslavskis

**Affiliations:** 1 Institute of Chemical Physics, University of Latvia, Jelgavas Street 1, LV-1004 Riga, Latvia; liga.avotina@lu.lv (L.A.); annija_elizabete.goldmane@lu.lv (A.E.G.); gunta.kizane@lu.lv (G.K.); 2 Institute of Solid State Physics, University of Latvia, Kengaraga Street 8, LV-1063 Riga, Latvia; lbikshe@cfi.lu.lv (L.B.); smits@cfi.lu.lv (K.S.); 3 Institute of Biomedical Engineering and Nanotechnologies, Riga Technical University, 6B Kipsalas Street, LV-1048 Riga, Latvia; marina.romanova@rtu.lv (M.R.); hermanis.sorokins@rtu.lv (H.S.); aleksandrs.vilkens@rtu.lv (A.V.); 4 Joint Stock Company “ALFA RPAR”, 140 Ropazu Street, LV-1006 Riga, Latvia

**Keywords:** planar field emission microtriode, tungsten, silicon dioxide, field emission, field emission cathode, electrical properties

## Abstract

This study examines the electrical properties and layer quality of field emission microtriodes that have planar electrode geometry and are based on tungsten (W) and silicon dioxide (SiO_2_). Two types of microtriodes were analyzed: one with a multi-tip cathode fabricated using photolithography (PL) and the other with a single-tip cathode fabricated using a focused ion beam (FIB). Atomic force microscopy (AFM) analysis revealed surface roughness of the W layer in the order of several nanometers (Ra = 3.8 ± 0.5 nm). The work function values of the Si substrate, SiO_2_ layer, and W layer were estimated using low-energy ultraviolet photoelectron emission (PE) spectroscopy and were 4.71 eV, 4.85 eV, and 4.67 eV, respectively. The homogeneity of the W layer and the absence of oxygen and silicon impurities were confirmed via X-ray photoelectron spectroscopy (XPS). The PL microtriode and the FIB microtriode exhibited turn-on voltages of 110 V and 50 V, respectively, both demonstrating a field emission current of 0.4 nA. The FIB microtriode showed significantly improved field emission efficiency compared to the PL microtriode, attributed to a higher local electric field near the cathode.

## 1. Introduction

Advances in micro- and nanotechnology have led to a renewed interest in vacuum electronics [1,2,3,4,5]. Vacuum electronic devices have several advantages over semiconductor devices, making them the preferred choice for some applications. For example, vacuum devices are widely used in wireless communications and high-speed data transmission systems due to their ability to operate at high power levels and high frequencies [6,7,8,9]. Vacuum devices also have a high radiation tolerance, making them suitable for use in aerospace technology and other environments with high radiation activity levels, such as particle accelerators or radiation sources and detectors [10,11,12,13,14].

Microtriodes with field emission cathodes are one of the key components of vacuum electronic circuits and are used to amplify and control electrical signals. It is generally known that field emission cathodes have several advantages over thermionic cathodes, including longer life, reduced size and weight, higher power efficiency, and faster response time. However, they are more complex to manufacture because they require a very precise and controlled manufacturing process to achieve high tip sharpness. Field emitters for microelectronics are usually fabricated as vertically standing structures, such as sharp cones, tips, nanotubes, etc. [15,16,17,18,19,20]. To fabricate these structures, planar semiconductor technologies are often complemented by nanotechnologies. This complicates the design of field emission devices and increases their production costs. To reduce the costs, the planar geometry of the cathodes can be utilized.

This study compares the current and field emission characteristics of two types of microtriodes with the planar geometry of their electrodes. The first microtriode, referred to as a photolithography microtriode (PL), has a multi-tip cathode and is fabricated using planar semiconductor technologies and photolithography. The second microtriode, referred to as a focused ion beam microtriode (FIB), has a single-tip cathode and is fabricated using a focused ion beam. The choice of these two microtriodes allows us to study the influence of cathode design on the electrical characteristics of the microtriodes.

The choice of cathode type for the user will depend on the specific requirements of the application. Multi-tip cathodes have potentially higher-emission current density, lower threshold voltage, and greater resistance to damage. Multi-tip cathodes are generally more reliable than single-tip cathodes because there is a probability of electron emission from at least one of the tips, even if some of the tips are damaged or contaminated. On the other hand, the single-tip configuration allows for a more compact microtriode structure, which is an advantage in applications where size constraints are critical. Single-tip cathodes may also be preferred in applications where a high degree of special resolution is required, which is provided by the small size and sharpness of the emitting tip.

Furthermore, we evaluate the quality of the layers of the fabricated microtriodes by analyzing their surface roughness, elemental composition, and work function. The thermal properties and infrared spectroscopic analysis of the microtriode layers have been described in other works [21,22].

## 2. Materials and Methods

**Figure 1:**
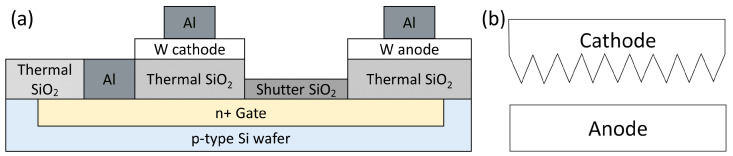
Schematic diagram of the PL microtriode: (**a**) microtriode layers; (**b**) mutual arrangement of the multi-tip cathode and rectangular anode, view from the top.

A schematic of the PL microtriode layers is shown in Figure 1a. To obtain the n+ gate layer, the surface of a p-type Si wafer was doped with phosphorus. Next, the oxidation process was performed at a temperature of 1130 °С to obtain a 0.6 μm thick thermal SiO_2_ layer. A 0.2 µm thick tungsten (W) layer was then deposited on the thick thermal SiO_2_ using DC magnetron sputtering. The deposition parameters included an argon atmosphere, a current of 150 mA, a pressure of 5 × 10^−3^ mBar, a temperature of 250 °C, and a deposition time of 3 min. The resulting resistance of the W layer was 3.8 ohm/square. The W layer was then etched to form the cathode and anode electrodes. Next, the thick oxide layer was etched to a thickness of 0.2 μm to obtain the gate oxide. Finally, windows were opened to make contact with the gate, and the aluminum (Al) wiring was formed.

**Figure 2:**
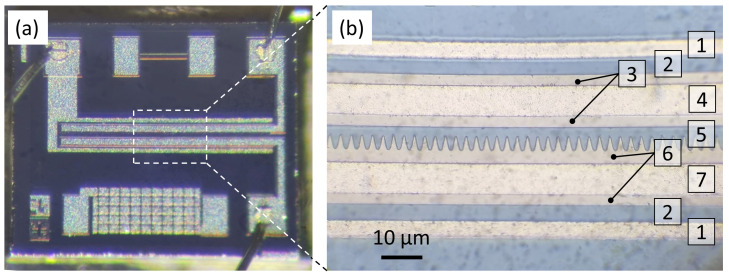
Optical microscope images of the PL microtriode: (**a**) image of the entire chip surface; (**b**) magnified view of the microtriode electrodes. Annotations in (**b**) correspond to the following layers: (1) Al connections to the gate; (2) thermal SiO_2_; (3) anode; (4) Al connection to the anode; (5) shutter SiO_2_; (6) cathode; (7) Al connection to the cathode.

The cathode of the PL microtriode has multiple tips oriented horizontally toward the anode, as shown in Figure 1b. The anode has a rectangular shape. The cathode tip angle is 22.6°, and the distance between the tips is 2.4 μm. There are 120 tips in total. The distance between the cathode and anode is 2 μm. Optical microscopy images of the fabricated PL microtriode are shown in Figure 2.

**Figure 3:**
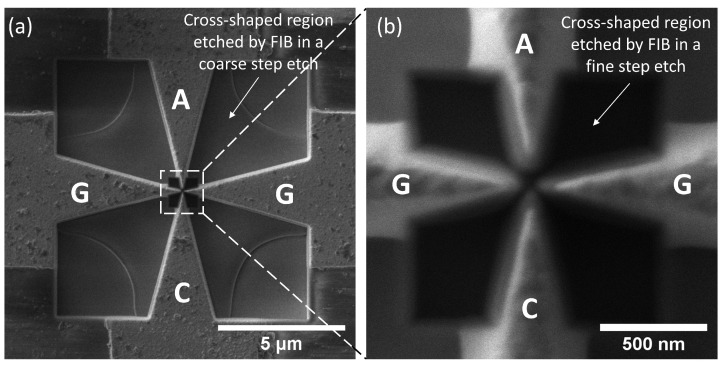
SEM images of the microtriode fabricated using FIB at different magnifications: (**a**) large-scale image; (**b**) close-up of the microtriode central area. Annotations: anode (A), cathode (C), two gate (G) electrodes.

Figure 3 shows scanning electron microscope (SEM) images of the second type of microtriode fabricated using a two-step FIB etching technique. The microtriode has a double-gate configuration with a cathode–anode distance of 100 nm, a gate-to-gate distance of 90 nm, and a cathode-to-gates distance of 15 nm. The gates, cathode, and anode electrodes have a taper angle of 30°. The radius of curvature of the anode electrode is 20 nm. A schematic diagram and the dimensions of the FIB microtriode are shown in Figure S1.

To fabricate the microtriode, a cross-shaped blank was first prepared using planar semiconductor technologies to form W bridges that connect the cathode, anode, and gate electrodes. FIB was then used to cut these bridges and create a gap between the electrodes. To prepare the blank, a 1.5 μm thick SiO_2_ layer was first grown on a p-type Si wafer through thermal oxidation at 1130 °С. A 0.2 µm thick layer of W was then deposited on the grown oxide using DC magnetron sputtering, using the same parameters as for the fabrication of the PL microtriode. To create the gap between the microtriode electrodes, a two-step FIB etching process was performed. In the first step, coarse structure etching was performed on a 10 × 10 µm area using a 30 kV and 1.2 nA ion beam, which reduced the connection area of the W layer. This etched region is shown in Figure 3a. Subsequently, a fine structure etching was performed on a 1.2 × 1.2 µm area using a 30 kV and 26 pA ion beam. The region etched in a second step is shown in Figure 3b. The fabrication of the microtriode was carried out using a Helios 5 UX dual-beam microscope (Thermo Scientific, Waltham, MA, USA).

To control the quality of the microtriode layers (W and SiO_2_), reference samples were prepared simultaneously with the fabrication of the microtriodes. These reference samples consisted of similar layers deposited on p-type Si wafers. The quality of the layers was analyzed in terms of their elemental composition, surface roughness, and work function.

The elemental composition of the W layer was characterized using X-ray photoelectron emission spectroscopy (XPS). The measurements were performed with a ESCALAB Xi+ spectrometer (Thermo Scientific, Brno, Czech Republic). The base pressure in the analytical chamber was less than 2 × 10^−7^ Pa. Monoatomic Ar+ ions with an energy of 3000 eV were used to etch the surface for depth profiling. The raster size was 1 × 1 mm. The atomic concentrations of W4f, O1s, and Si2p were measured after every 10 s of etching, with an estimated etching rate of 13.77 nm/s (Ta_2_O_5_ equivalent).

The surface roughness of the W layer was characterized using atomic force microscopy (AFM). An Solver P-47 PRO microscope (NT-MDT, Zelenograd, Moscow, Russia) and NSG10/Pt AFM probes (TipsNano, Tallinn, Estonia) with a tip radius of 35 nm were used. AFM images were acquired with a scan size of 10 × 10 µm and processed using the Gwyddion software (version 2.63). Prior to the surface roughness analysis, the images were leveled using the mean plane subtraction method; then, the polynomial background was removed, and the minimum data value was shifted to zero.

The photoelectric work function of the fabricated layers was estimated using ultraviolet (UV) photoelectron emission (PE) spectroscopy. The measurements were performed in a vacuum of 10^−3^ Pa using a custom-made PE spectrometer. The PE was excited by a 30 W deuterium source (LOT-Oriel Europe, Darmstadt, Germany) emitting photons in an energy range of 4.13–6.20 eV (wavelengths from 295 to 200 nm). PE current was measured as a function of photon energy, and an MDR-2 UV monochromator (Lomophotonica, Saint Petersburg, Russia) with automatic scanning was used to select the wavelengths. The emitted photoelectrons were detected using an SEM-6M secondary electron multiplier (VTC Baspik, Vladikavkaz, North Ossetia-Alania, Russia), which was connected to a custom-made preamplifier, a Robotron 20046 radiometer (VEB Robotron-Meßelektronik, Dresden, Germany), and an M8784 counting board (Hamamatsu Photonics K.K., Shizuoka, Japan). The uncertainty in the photon energy measurement was within ±0.03 eV. To determine the work function, the low-energy region of the PE spectrum was analyzed by extrapolating the measured PE current to zero.

**Figure 4:**
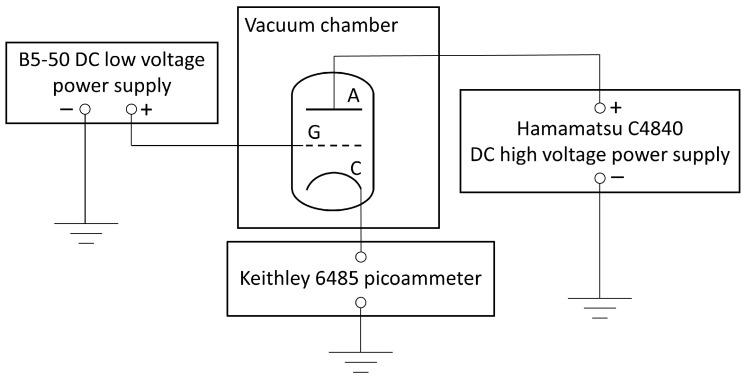
Schematic of the experimental setup for measuring the electrical parameters of the fabricated microtriodes.

The electrical parameters of the fabricated microtriodes were measured in a custom-made vacuum chamber at a pressure of 5 × 10^−5^ Pa. A schematic diagram of the experimental setup for testing the electrical parameters is shown in Figure 4. The potentials were applied to the microtriode electrodes using a B5-50 DC power supply (JSC “Nizhny Novgorod plant RIAP”, Nizhny Novgorod, Russia) and a C4840-02 high voltage power supply (Hamamatsu Photonics K.K., Shizuoka, Japan). The current flowing between the cathode and anode was measured using a Keithley 6485 picoammeter (Tektronix, Beaverton, OR, USA).

**Figure 5:**
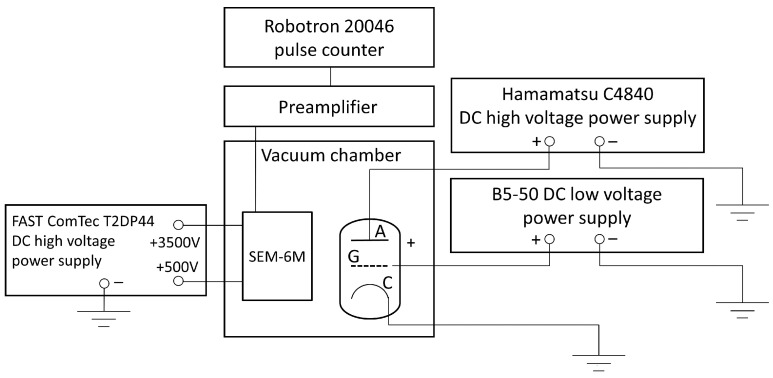
Schematic diagram of the setup for measuring field emission current through the cathode–anode vacuum gap using the electron counting method.

The field emission current passing through the vacuum gap between the cathode and anode was also detected using the electron counting method. In this case, the current was detected using an SEM-6M secondary electron multiplier (VTC Baspik, Vladikavkaz, North Ossetia-Alania, Russia), positioned above the microtriode in the vacuum chamber. The electron multiplier was connected to a custom-made preamplifier and a Robotron 20046 radiometer (VEB Robotron-Meßelektronik, Dresden, Germany). The accelerating potentials were applied to the electron multiplier using a T2DP-44 high voltage power supply (FAST ComTec Communication Technology GmbH, Oberhaching, Germany). The measurements were performed according to the setup shown in Figure 5.

## 3. Results

The W layer had an average surface roughness (*Ra*) and root-mean-square roughness (RMS) of 3.8 ± 0.5 nm and 0.8 ± 0.1 nm, respectively, as measured using AFM. The low roughness of the emitting layer in the order of a few nanometers indicates a good fabrication quality, since the roughness of this layer should not exceed the size of the electron-emitting part of the cathode. In addition, the low roughness of the W layer reduces the occurrence of surface defects, thereby improving the electron emission properties and reducing the possibility of electron scattering [23,24]. 

The photoelectric work function was measured to be 4.71 ± 0.08 eV for the p-type Si substrate, 4.85 ± 0.11 eV for the SiO_2_ layer, and 4.67 ± 0.06 eV for the W layer. The work function of the emitting layer must be lower compared to the materials of the other layers surrounding the cathode.

**Figure 6:**
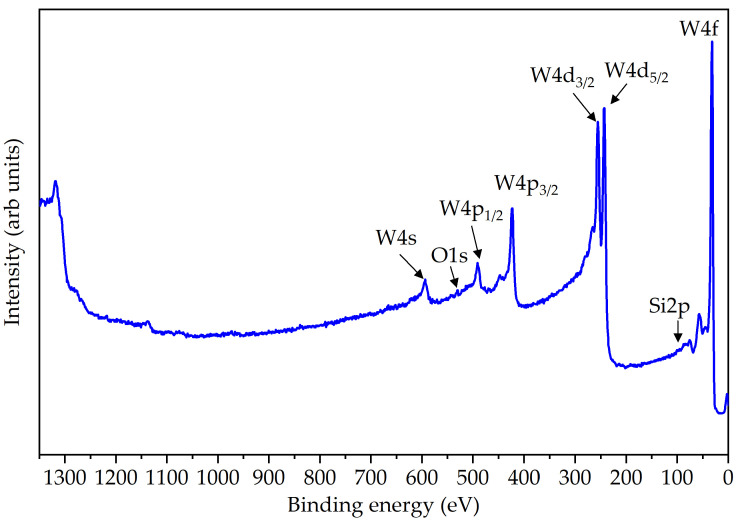
XPS survey spectrum of the W layer deposited on a Si/SiO_2_ substrate; individual core levels of tungsten and the absence of oxygen and silicon signals are marked.

Figure 6 presents the XPS survey spectrum of the W layer. Prior to the measurements, the surface of the W layer was pre-etched with Ar+ ions for 10 s inside the XPS spectrometer chamber to remove possible surface carbon contamination. The XPS database from the reference [25] was used to identify the observed spectral features. The spectrum showed the presence of only W peaks, indicating the absence of O and Si contamination in the W layer. The binding energies where O1s and Si2p signals would be expected are also marked in Figure 6 for reference.

The XPS depth profiling results of the W layer for the presence of tungsten, oxygen, and silicon are shown in Figure S2. The increase in Si2p and O1s signals in the depth profiles indicated that the W layer was removed after about 1000 s of etching, and the underlying SiO_2_ layer was reached. The presented depth profiling results demonstrated the absence of O and Si traces on both the surface and the bulk of the tungsten layer, indicating its homogeneity.

The high-resolution W4f spectrum is presented in Figure S3. To eliminate any probable impact from surface oxides, the spectrum was obtained after the W layer was etched for 400 s. The positions of the detected peaks were analyzed using databases from references [26,27]. The peak at 31.2 eV corresponds to W 4f7/2, the peak at 33.4 eV corresponds to W 4f5/2, and the peak at 36.7 eV corresponds to W 5p3/2. The binding energies of the peaks confirm the presence of metallic tungsten.

This section further presents the theoretical background and experimental results used to evaluate the electrical characteristics of the fabricated microtriodes.

When an external voltage is applied to a metal cathode at an electric field strength of 10^5^ V/cm, the potential barrier height at the metal–vacuum interface decreases due to the Schottky effect [28]. If the field strength is further increased to 10^7^–10^8^ V/cm, the potential barrier height and width decrease so much that quantum mechanical tunneling becomes the dominant mechanism [29]. This leads to the emission of electrons into the vacuum, which is known as field electron emission. The relationship between the emission current density (*J*) and the electric field strength (*E*) between the electrodes in field electron emission is described by the Fowler–Nordheim equation [30]:

(1) $$J=\frac{e^{3}\cdot E^{2}}{8\cdot \pi \cdot h\cdot \varphi \cdot t^{2}\left(E,\varphi \right)}\cdot \exp\left[-\frac{8\cdot \pi \cdot {\left(2m\right)}^{1/2}\cdot \varphi^{3/2}\cdot \Theta \left(E,\varphi \right)}{3\cdot h\cdot e\cdot E}\right]$$

where:

*e*—the charge of an electron,
*ϕ*—the work function of the cathode material,
*m*—the mass of an electron,
*h*—Planck’s constant.

The functions *t*(*E*,*ϕ*) and *Θ*(*E*,*ϕ*) are special functions that account for the influence of mirror image forces on the reduction in the triangular potential barrier, which affects the current value in field electron emission.

For practical purposes, the value of the function *t*(*E*,*ϕ*) can be assumed to be equal to 1. The values of both *t*(*E*,*ϕ*) and *Θ*(*E*,*ϕ*) have been tabulated in previous research [31].

Based on the experimental observation of field electron emission from metals, it is assumed that the electric field strength near the cathode surface is equal to or greater than 10^7^ V/cm [28]. To achieve this, cathodes with non-uniform fields are commonly used, often employing tips with an extremely small radius of curvature. The electric field strength (*E*) at the apex of the tip is directly proportional to the applied voltage (*U*):

*E* = *β*∙*U*, (2)

where *β* is the field enhancement factor [32]. The field enhancement factor is determined by solving the corresponding electrostatic problem and depends only on the geometry and dimensions of the cathode–anode system [33].

In an actual experimental setup, direct measurements of the current density (*J*) or the area of the electron-emitting surface (*S*) are not possible. Instead, the total current (*I*) is measured, which is the product of the current density and the electron-emitting surface area:

*I* = *J*∙*S*, (3)

By utilizing Equations (2) and (3), substituting the values of the physical constants, and taking the logarithm of Equation (1), one can rewrite this equation in a form convenient for processing experimental data:

(4) $$lg\left(\frac{I}{U^{2}}\right)=10.188+lg\left(\frac{S\cdot \beta^{2}}{\varphi \cdot t^{2}\left(E,\varphi \right)}\right)-\frac{0.297\cdot \varphi^{3/2}\cdot \Theta \left(E,\varphi \right)}{\beta }\cdot \frac{1}{U}$$

where:

I—the field electron emission current in A,
*U*—the applied voltage in V,
*E*—the electric field strength in V/Å,
*ϕ*—the work function in eV,
*β*—the field enhancement factor in 1/Å,
*S*—the emitting surface area in cm^2^.

The value of *β* can be determined by analyzing the slope of the linear part of the lg(*I*/*U*^2^) dependence on 1/*U*. Also, the intersection point of this straight line with the lg(*I*/*U*^2^) axis gives the area of the electron-emitting surface (*S*).

Equation (4) shows that the field enhancement factor is inversely proportional to the slope:

(5) $$\beta =\frac{0.297\cdot \varphi^{3/2}\cdot \Theta \left(E,\varphi \right)}{\mathrm{slope}}$$

**Figure 7:**
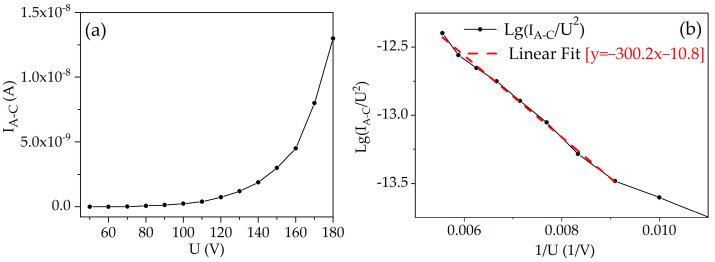
Anode–cathode current (*I_A-C_*) characteristics for the PL microtriode: (**a**) *I_A-C_* dependence on the anode–cathode voltage (*U*); (**b**) Fowler–Nordheim plot.

Figure 7a shows the relationship between the anode–cathode current and the voltage across the anode and cathode. The current dependence on the applied voltage exhibits exponential behavior. The same relationship is represented in Figure 7b using Fowler–Nordheim coordinates. The turn-on voltage for the field emission was found to be 110 V, with a current of 0.4 nA. Beyond this voltage threshold, the dependence exhibits a linear pattern, indicating the onset of field emission current.

**Figure 8:**
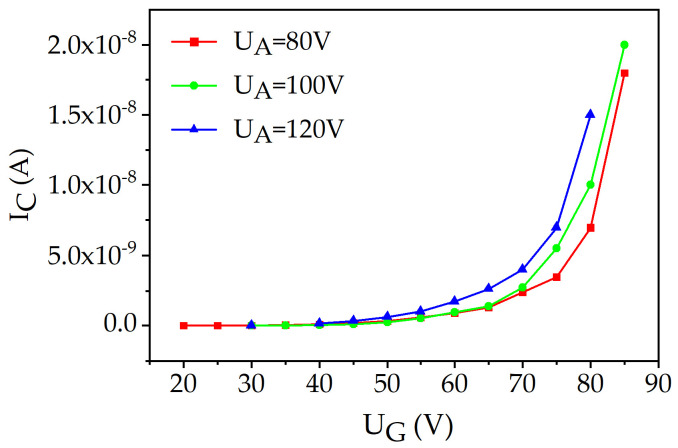
Dependence of the cathode current (*I_C_*) on the gate electrode potential (*U_G_*) at different anode potentials (*U_A_*) for the PL microtriode.

Figure 8 shows the relationship between the cathode current (*I_C_*) and the potential of the gate electrode (*U_G_*) at various anode potentials (*U_A_*). As observed in the figure, increasing the anode potential leads to a corresponding increase in the cathode emission current. This can be attributed to the enhanced electric field strength in the anode–cathode gap.

To validate the occurrence of field emission current across the vacuum gap between the cathode and anode in the PL microtriode, electron current measurements were conducted using the electron counting method. The measurements were carried out following the setup shown in Figure 5. A voltage of 180 V was applied between the anode and cathode, and the current of 1000 electrons per second was recorded.

**Figure 9:**
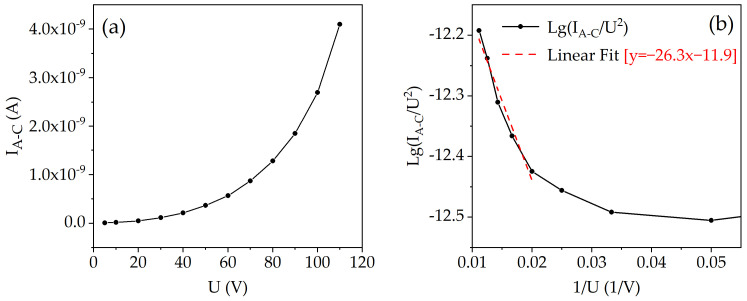
Anode–cathode current (*I_A_*_-*C*_) characteristics for the FIB microtriode: (**a**) *I_A_*_-*C*_ dependence on the anode–cathode voltage (*U*); (**b**) Fowler–Nordheim plot.

The electrical measurements were carried out on the FIB microtriodes according to the same procedure as for the PL microtriodes (schematics in Figure 4). The relationship between the anode–cathode current and the voltage across the anode and cathode is shown in Figure 9a, and the corresponding Fowler–Nordheim plot is shown in Figure 9b. The turn-on voltage for the FIB microtriode was found to be 50 V, with a current of 0.4 nA.

**Figure 10:**
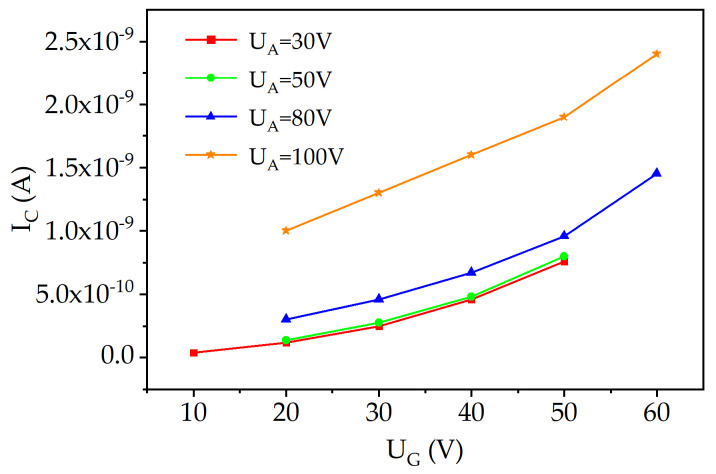
Dependence of the cathode current (*I_C_*) on the gate electrode potential (*U_G_*) at different anode potentials (*U_A_*) for the FIB microtriode.

Figure 10 shows the relationship between the cathode current (*I_C_*) and the potential of the gate electrode (*U_G_*) at various anode potentials (*U_A_*) for the FIB microtriode. When comparing Figure 8 and Figure 10, it can be observed that the anode potential has a more pronounced effect on the dependence of *I_C_*(*U_G_*) for the FIB microtriode. 

The local electric field strength near the cathode can be estimated using Equation (2), where the electric field strength is the product of the applied voltage and the field enhancement factor. The latter is inversely proportional to the slope according to Equation (5). The Fowler–Nordheim plots in Figure 7b and Figure 9b show that the slope for the PL microtriode and the FIB microtriode was 300.2 and 26.3, respectively. Hence, it is inferred that the local electric field near the cathode of the FIB microtriode is one order of magnitude higher than that of the PL microtriode. This result can be attributed to the significantly reduced anode–cathode distance in the FIB microtriode, which is an order of magnitude smaller than that of the PL microtriode.

## 4. Conclusions

Two types of planar vacuum microtriodes and the fabrication quality of their layers were investigated. The PL microtriode had a multi-tip cathode and was fabricated using planar semiconductor technologies and photolithography. The FIB microtriode had a single cathode and was fabricated by FIB. Surface roughness analysis showed that the W layer of the microtriodes exhibited roughness values in the range of several nanometers, indicating good fabrication quality with reduced probability of surface defects. The photoelectric work function values of the Si substrate, SiO_2_ layer, and W layer were estimated. XPS analysis of the W layer confirmed the absence of oxygen and silicon impurities, highlighting the homogeneity of the W layer.

Using the secondary electron multiplier and electron counting method, it was demonstrated that the current passes through the vacuum gap between the anode and cathode, confirming the occurrence of field electron emission. The Fowler–Nordheim equation was used to describe the relationship between the emission current density and the electric field strength. The analysis also showed that in the case of the FIB microtriodes, the local electric field near the cathode significantly exceeds that of the PL microtriodes, which was attributed to the smaller anode–cathode distance. This resulted in a significantly improved field electron emission efficiency for the FIB microtriodes. Also, the effect of the anode potential on the dependence of the cathode current on the gate electrode potential was more pronounced in the FIB microtriodes. The obtained results highlight the improved efficiency and performance of the FIB microtriodes in terms of field electron emission.

## Data Availability

The data presented in this study are available on request from the corresponding author.

## Supplementary Materials

The following supporting information can be downloaded at: https://www.mdpi.com/article/10.3390/ma16175781/s1, Figure S1: Configuration of the cathode (C), anode (A), and two gate electrodes (G) in a microtriode fabricated using FIB. Dimensions are in nanometers, not to scale; Figure S2: XPS depth profiling of a 200 nm thick W layer deposited on a Si/SiO_2_ substrate, depth profiling spectra for tungsten, oxygen, and silicon are shown; Figure S3: W 4f5/2, W 4f7/2, and W 5p3/2 high-resolution XPS spectrum for tungsten metal.
