# Supplementary material for: Tungsten–SiO2–Based Planar Field Emission Microtriodes with Different Electrode Topologies
Source: Materials (Basel). 2023 Aug 24;16(17):5781. doi: 10.3390/ma16175781 (PMC10488438; doi:10.3390/ma16175781)
Supplement: Supplementary file 1 [file materials-16-05781-s001.zip › materials-2507873-supplementary.pdf]

Supplementary Materials

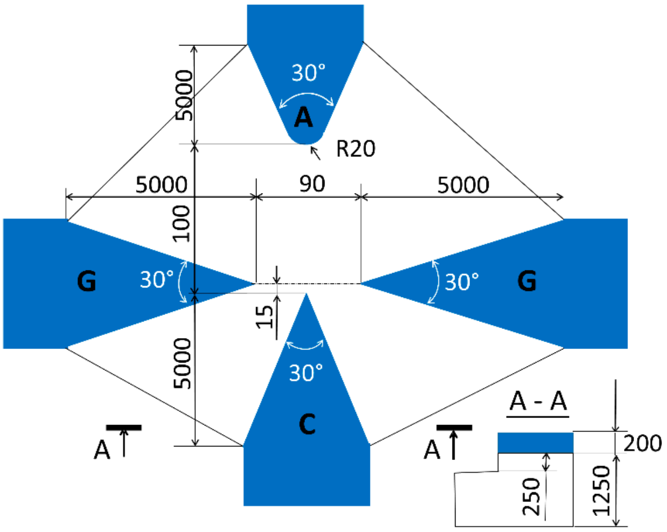

Figure S1. Configuration of the cathode (C), anode (A), and two gate electrodes (G) in a microtriode fabricated using FIB. Dimensions are in nanometers, not to scale.

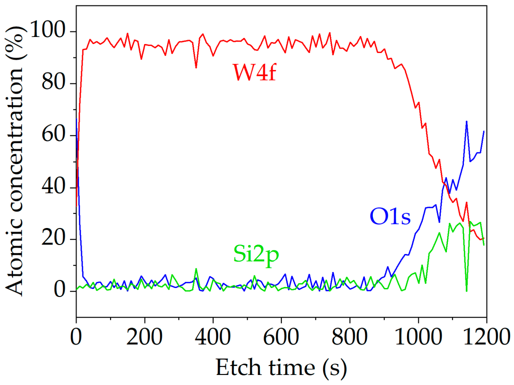

Figure S2. XPS depth profiling of a 200 nm thick W layer deposited on a Si/SiO_2 substrate, depth profiling spectra for tungsten, oxygen, and silicon are shown.

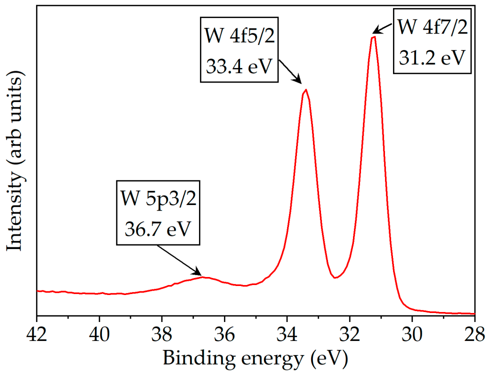

Figure S3. W 4f5/2, W 4f7/2, and W 5p3/2 high-resolution XPS spectrum for tungsten metal.
